# Jan of Jenštejn’s *De bono mortis*: Reconstructing a Sceptical Patient’s Travel Narrative

**DOI:** 10.1093/shm/hkaf029

**Published:** 2026-03-09

**Authors:** Patrick Outhwaite

**Affiliations:** Department of English Language and Culture, University of Groningen, 9712 EK Groningen, The Netherlands

**Keywords:** Jan of Jenštejn, medieval, Bohemia, medical scepticism, frequent communion

## Abstract

While travelling across Europe, Jan of Jenštejn, archbishop of Prague (1379–96; d. 1400) suffered many illnesses. As recorded in his personal correspondence and the last work in his oeuvre, *De bono mortis*, Jenštejn frequently fell ill and encountered what he deemed to be incompetent physicians who were ignorant of how to treat his complexion. Coloured by these experiences, Jenštejn devoted a significant portion of his *De bono mortis* to criticising medicine and voicing his scepticism. He claimed to reject all forms of earthly medicine and placed his trust solely in frequent reception of the Eucharist as a sovereign treatment for spiritual and bodily health. As this article demonstrates, Jenštejn’s narratives of illness not only reveal the rhetoric surrounding his medicalised sacramental theology, but also offer a means of conceptualising the ways in which travel complicated experiences of illness and treatment.

Late in 1390, Jan of Jenštejn, archbishop of Prague (1379–96; d. 1400) travelled to Rome for the jubilee and to petition Pope Boniface IX to finish the process of establishing the feast of the Visitation of the Blessed Virgin Mary.^1^ On his return to Prague, he was struck by a life-threatening illness that left him deaf, dumb, blind and paralysed. Jenštejn describes his brush with death in detail:

at a distance of only three miles from the city of Prague, suddenly a languor seized me with a terrible fever of a natural one day interval, that kept me shaking wretchedly, and it laid me low even to the point of death. After the most violent pain and grave infirmities consumed me for many days, with physicians despairing of my life and others similarly frustrated in their hope, namely, my friends and those close to me, I, too, in the same manner, did not think that I ought to live any longer […]. Then I lay bent at the knees upon a pillow, but from the excessive debilities possessing me, rose a certain frenzy, which when I realised it had increased notably, I began to rise and was hastened to the presbyter […]. But when the presbyter delayed in giving the saving host, *darkness had covered me* [Psalms 54: 6] and I was not able to see with open eyes; I became thoroughly deaf and dumb and as those bystanders and those who held me claimed, many of my bones seemed to clatter and creak like the dead, and the signs of a man about to die were noticeably visible to others on the outside, inside truly my soul was tormented with indescribable pain.^2^

From this account, it appears as if the illness arose without cause, yet it seems Jenštejn’s long journey exacerbated existing problems of chronic ill health. According to his confidant and biographer Petr Klarifikátor of Roudnice (fl. 1382–1406), Jenštejn wore a sack cloth under his clothes on his journey to and from Rome. This sack cloth was an additional means of enacting penance and a reminder of the penitential significance of pilgrimage. Klarifikátor explains that the sack cloth was also a contributing factor to his malady, as he implies that the intense skin irritation left him more vulnerable to ill health.^3^

Jenštejn’s account, much like Klarifikátor’s biography, is far from objective, imitating hagiographic conventions in presenting himself as a suffering saintly figure.^4^ A large number of saints experienced intense illness and injury, and hagiographic texts emphasised suffering as a core aspect of the religious life. In fact, for many saints, infirmity served as an early indicator of their later saintly status.^5^ As is also typical for hagiographical accounts, physicians and members of the clergy were clueless of how to treat Jenštejn’s sudden onset of paralysis and the dampening of his senses.^6^ It was only after he took communion that he was eventually healed:

Meanwhile, I happened to recall to my memory that I ought still to take communion; for in my permissible pressures, I had forgotten to take communion. Therefore, I said to myself: ‘Although my eyes are dim [cf. Genesis 27: 1], so that I may not be able to look upon my Lord, at least I will open my mouth to receive this venerable sacrament.’ And thus my mouth opened and reverently accepted the body of the Lord, *attracting the spirit* [Psalms 118: 131]. And at that moment my eyes were opened and I saw the presbyter waiting in front of the altar with the Eucharist, with the rest standing around him covered in tears. And when I saw the living bread, which *gives life to the world* [John 6: 33; 41], I noticed that the bond that seized my lips loosened and I began to say: ‘my Lord the King [1 Kings 24: 9].’ But the priest soon extended the Lord’s body to me, and at once all pain and weakness left me, except for only a slight remaining trace.^7^

Jenštejn draws two conclusions from this illness and his recovery. First, his symptoms seemed to be caused as much by sin as by his physical state. In fact, illness and sin could not be untangled in his discourse of health; a festering sin could cause a physical malady just as readily as any other factor. This is not to suggest that the Archbishop was a sinful man, but rather that his ascetic life forced him to consider any and all transgressions, no matter how slight, along with the corruptible nature of the human body to be causes for great lamentation. Still, illness was not simply an indicator of humanity’s sinful nature, but could also be a sign of divine favour. Jenštejn’s complex rendering of sin and illness is representative of a broader trend in medieval Europe in which illness could at once be a cause for sorrow and be welcomed and valorised.^8^ Throughout his works, Jenštejn viewed his illnesses as tools for his penitential development, serving as a physical manifestation of contrition and as proof of his ascetic piety. Jenštejn’s concept of spiritual infirmity led to the second conclusion, that the Eucharist was a sovereign cure for all manner of maladies. For Jenštejn, earthly medicine was entirely impotent and it was not until he received the communion wafer that he was able to recover. Hence, Jenštejn proposed a theory of health in which illness could only be cured through a sacramental medicine, namely, frequent reception of communion. Throughout his life Jenštejn treated his illnesses and recoveries as proof of his sanctity, and he encouraged others to adopt a similar devotional attitude towards health and healing.

The episode outside of Prague inspired Jenštejn to write his longest work, *De bono mortis* (composed between 1392 and 1393), which presents frequent reception of communion as the most effective medicine.^9^ Yet prior to 1390, the clergy in Prague, led by Archbishop Jenštejn himself, took measures to restrict the frequency with which the laity took communion. These measures were made on the back of a rise in a grassroots movement led by the likes of Jan Milíč of Kroměříž (d. 1374) and Matěj of Janov (d. 1393) to encourage daily reception of the sacrament of the altar.^10^ To counter this popular movement, a clerical synod in 1388 declared that communion could be taken a maximum of once per month by members of the laity.^11^ These restrictions were part of a broader policy instituted by the Church in Prague to clamp down on a supposed increase in religious dissent in the city and its surrounding area.^12^ After his brush with death in 1390, however, Jenštejn reversed his position on frequent communion. A synod of 10 October 1390 allowed frequent reception of the sacrament if taken following confession. On 16 June 1391, Jenštejn declared before another synod that ‘true penitents, provided they confess and are penitent and worthy of the sacrament, may receive it as often as they desire.’^13^

*De bono mortis* was written on the back of Jenštejn’s turn towards frequent communion as an ideal practice, and at each stage of the text, the healing power of the Eucharist is held up as the only effective form of medicine. Drawing on patristic and biblical authorities, Jenštejn attempts to define and document death in its literal and allegorical guises. Much like the treatise of the same name by St Ambrose of Milan (d. 397), *De bono mortis* distinguishes between the death of sinners and the figurative death to the world of the righteous.^14^ Jenštejn presents personal examples of illness, which follow a similar pattern to narratives of miraculous healing in saints’ lives: the patient has a mysterious illness that baffles all experts until he or she is healed by God.^15^ As Gottfried Vielhaber explains, Jenštejn’s work is not meant to be a scholastic tractate that rationally outlines his theological stances, rather it is part of a trend of devotional works dealing with the topic of death that draws on personal experience.^16^ Still, Pavlína Rychterová has shown that Jenštejn’s account of his piety and intense ascetism had political consequences that went beyond the personal, as the text was aimed at pressuring the Church into enacting reform. Indeed, the work is part of a trend of texts produced by reformists who emphasised the importance of a personal engagement with the divine.^17^

These devotional and political elements make *De bono mortis* a difficult text to classify. As Ruben E. Weltsch explains, it combines personal reflection, devotional instruction, sacramental theology and various tropes associated with hagiography.^18^ This article further refines our reception of *De bono mortis*, demonstrating that the text intertwines its biographical, devotional and theological themes with a critique of scholastic medicine. The text contributes to a rich tradition of condemnations of the shortcomings of medieval physicians that emerged from the mid-thirteenth century, as critics condemned what they saw as an unhelpful focus at medical faculties on theory at the expense of pragmatic or instrumentalist treatment.^19^ In the fourteenth and fifteenth centuries, this preference for practical medicine and pharmacology was adopted by certain university masters, but criticism of scholastic medicine and its reputation for logic-chopping continued.^20^ Jenštejn adds to this criticism, and in fact appears to go further than many of his contemporaries in rejecting both practical and theoretical medicine.

Jenštejn’s accounts of illness from *De bono mortis* can be supplemented with evidence from his collection of seventy-seven letters from c. 1370–1391, preserved in Prague, Státní ústřední archiv č. 2449, ff. 1^r^–54^r^.^21^ Zdeňka Hledíková has demonstrated that the manuscript was likely created under the direct supervision of Jenštejn, who polished the epistulae before handing them over to the scribe for copying.^22^ As we will see, the details recorded in the epistulae do not always match Jenštejn’s later account. In all probability he altered the experiences in *De bono mortis* to fit his theological needs; after all, it was not until 1390 that he began his campaign of promoting frequent reception of communion. Hence, Jenštejn revises his personal history of illness to suit his present needs.^23^

What makes Jenštejn’s narratives so useful in the study of the history of medicine, is that usually examples of illness and treatment are told from the view of the practitioner, but in Jenštejn’s writings, we read the experience of a patient first hand. Jenštejn’s illnesses were complicated by the fact that he travelled throughout Europe and was treated in foreign lands by unfamiliar physicians. During his travels, in which he found himself at the mercy of numerous physicians from different lands, he established a theory of spiritual health in which the Eucharist was a sovereign preventative medicine and remedy for all ailments. Of course, as already established, in *De bono mortis* Jenštejn had a clear agenda in pushing frequent communion at a time in which other Bohemian Church authorities were pushing back against all forms of religious dissent, and thus we cannot take his narratives of health at face value. In fact, the resistance of the Bohemian Church to frequent communion may have pushed Jenštejn towards a more extreme position in rejecting all forms of earthly medicine.

Nevertheless, to state that Jenštejn entirely opposed all forms of medicine would be to read him too literally. As Iona McCleery states in relation to the cult of the Portuguese Dominican friar and medical master St Gil de Santarém (d. 1265), religion and medicine were made to ‘compete with each other’ in certain devotional contexts.^24^ It is recorded in the *Vitae fratrum ordinis praedicatorum* (composed c. 1260) that Gil de Santarém supposedly urged the sick to avoid medical treatment because Christ was more powerful than Galen. This statement set up a binary that was proven through theological narratives of healing in which Christ cured maladies that were unable to be remedied by earthly medicines. Yet in practice, religion and medicine were connected.^25^ Gil de Santarém did not replace all forms of medicine with faith, and he acknowledged the ways in which earthly medicine was necessary and complimentary to spiritual healing. Similarly, Jenštejn’s *De bono mortis* is a devotional text, and his denunciation of medicine must be understood as an established topos of the genre. By rebuking medicine and its authorities, he attempted to demonstrate the ways in which religion was the underpinning of all knowledge and that one’s health was dependent on God’s will. Indeed, his text draws from a long tradition of asceticism, the *ars moriendi* and the valorisation of illness and suffering.^26^

Often in hagiographical works, physicians and surgeons are introduced to illustrate the ways in which the illness and cure were beyond the earthly, yet this is not the case in the texts examined in this article.^27^ Jenštejn’s scepticism did not stop him from seeking out medical treatment. Evidence from *De bono mortis*, his personal correspondence and the account of his life written by Petr Klarifikátor all reveal that Jenštejn consulted numerous physicians. Further, Jenštejn studied medicine at Montpellier and throughout his works uses medical theories to diagnose his various ailments. Even at his most sceptical moments, Jenštejn invokes the very same authorities and theories of medicine that he condemned as useless.

This article investigates how Jenštejn’s travels and changing sacramental theology influenced his thinking about medicine. I contend that his scepticism of medicine was a constant throughout his life, with its roots in his early experiences of travel. In *De bono mortis*, he recalls these experiences of illness as a means of justifying frequent communion as a medicine, but his scepticism was already present. His narratives of illness were complicated by his travels, as he used ideas of unfamiliar complexions to justify painting the practitioners he encountered as clueless and impotent in comparison with Christ the divine Physician. In *De bono mortis*, Jenštejn pushes the polemical position of the superiority of Christ’s healing to the extreme. He asserts that the ideal earthly physician should not rely on outdated medical authorities or their remedies, but should accept the healing nature of the Eucharist even if they cannot rationally explain it through medical theories; his narratives of sickness in foreign lands prove the efficacy of frequent communion as the most effective cure for all manner of illnesses.

## Jan of Jenštejn in Montpellier: Climate and Complexions

Throughout his life, Jan of Jenštejn was a sickly figure who frequently suffered from ailments, which were, in the estimation of Petr Klarifikátor, key to his turning to an ascetic life.^28^ For example, during his time as a student in Montpellier, from 1375 to 1376, Jenštejn endured a particularly severe malady that rendered him bedridden.^29^ He complained in a letter to a friend, Beneš of Hořovice (Benessio de Horssow), canon and archdeacon of Horšovský Týn, that his illness arose due to the hot climate of the city:

For among other things, Phoebus in his passion proffers life-giving heat to such an extent that, with the humour spreading within, the perspiration is driven outward in a large flow, such that with my hand I frequently wipe my face, which is drenched with constant perspiration.^30^

Jenštejn acknowledged that the Mediterranean heat was essential for the abundance of wheat and wine produced in Montpellier, yet Phoebus provided too much sunlight and heat for one unaccustomed to this climate. Jenštejn’s complexion was not used to dealing with such intense heat and thus his body sought to compensate by perspiring excessively. The resulting illness caused by his imbalanced humours did not simply afflict his body, but also his mind, as he was unable to decide what to do about his illness:

Therefore, I do not know what to do with these troubles: I am not able to satiate [lit. provide for] thirst, nor to drive out the heat, nor to retrieve strength from my ailing purse, and thus completely enclosed by urgencies, as if placed in a trap, I do not know what option to decide upon, and from which I may doubtless be able to strengthen my constancy of mind; and thus many evils result from these issues, for the wandering mind drifts, much occupied in desires, and the body is worn out by weariness, so that I can neither concentrate properly on study nor, as if divided into several parts, attend to what I ought.^31^

Unable to decide whether to attempt to quench his thirst or to allow his body to cool through sweat, Jenštejn was in a particularly difficult spot. His difficulties were compounded by his dwindling finances, because he did not have the funds to consult another physician. Jaroslav V. Polc suggests that Jenštejn’s purse had been emptied by consulting multiple physicians, which is not entirely clear from the context.^32^ It seems odd for a man of his stature to be out of funds, and so the assertion may have been made for rhetorical purposes rather than reflecting reality. After all, the trope of the patient who had spent all their money on ineffective medical treatment was common in hagiographical writing.^33^ Regardless of the burden on his funds, without treatment he was unable to focus his mind, and while he continued to reside in such a torrid environment his body was unable to achieve humoral balance.^34^

After his illness in Montpellier, Jenštejn returned to study in Paris in 1376, a more amenable climate and temperature that was closer to that which he was used to in Prague, and indeed he maintained better health.^35^ Despite the benefits of moving to a different climate for one’s health, Jenštejn did not necessarily endorse travel to escape illness. In 1380, the same Beneš he wrote to from Montpellier, left Prague in search of a location with better air quality in order to escape the miasma of plague.^36^ When Jenštejn found out that Beneš had fled the city, he reprimanded him in a letter, explaining that it was precisely when a man attempted to escape death that he could not avoid it. God determined a specific length of life for each person that could not be altered or extended. Jenštejn urged him to return to Prague, even if his return should mean death, because a voluntary death was a kind of martyrdom.^37^ Thus, Jenštejn acknowledged the ways in which environmental factors, in this case air quality and miasma, could influence health, but a complete surrendering to God’s will informed all of his thinking.

Jenštejn’s connection between climate and health was not out of step with contemporary medical theory. Medical textbooks in university settings—such as Johannitius’s *Isagoge*, Galen’s *De complexionibus*, Haly Abbas’s *Liber Pantegni* and Avicenna’s *Liber canonis*—included sections on the differences between the complexions of people from hot and cold climates. This measuring of complexions and the effects of climate enabled medical authorities and their commentators to make general assumptions about peoples of different regions based on external factors as well as supposedly innate traits.^38^ Avicenna, for instance, distinguished between the complexions of southerners, a generic term used to refer to peoples of hot climates, and northerners, referring to those of cold climates, especially those from northern Europe. Northerners had hot complexions because their cutaneous pores were constricted by the cold temperatures. Since the pores were restricted, internal heat did not leave the body freely and it was retained in the organs. Southerners, by contrast, had open pores through which internal heat escaped the body. Heat was attracted by its like and thus the hot temperature outside the body encouraged internal heat to vent.^39^ Hence, as Avicenna determined, a balanced complexion did not have a uniform value, but differed depending on the environment of the patient.^40^ While some medical authorities suggested that a patient that was used to one climate could acclimatise to a different location if they spent an extended period there,^41^ for travellers the possibility of acclimatising was limited by the duration of their stay.^42^

Differences in complexion did not simply relate to how one coped with the climate. An unfamiliar complexion could pose a problem to a physician, as it would make the effects of his medicines unpredictable. Reflecting back on his time in Montpellier in his *De bono mortis*, Jenštejn explained that he eventually became so ill that he was taken under the care of one of the most renowned physicians of the late-medieval period, Jean Jacmé (Johannes Jacobi; d. c. 1384) the chancellor of the university in Montpellier:

And so this which I recount for the sake of learning happened to me when I was spending time in Montpellier, where there is the most exceptional *studium* of medicine in the world and where distinguished physicians are found. In that very place I became violently ill for approximately a full week. Among other very notable physicians, one named Jean Jacmé visited me—whom afterward Charles, the King of the French at Paris, retained in order to be under his care—while I remained in that *studium* because of its distinction.^43^

It is certainly conceivable that Jenštejn could have been treated by such a high-­profile physician as Jean Jacmé. After all, he mixed in courtly circles, notably that of King Charles V of France (d. 1380).^44^ On the other hand, Jenštejn may have chosen Jacmé for the story because he was one of the most prominent physicians at Jenštejn’s time of writing—a time in which Jacmé was chancellor of the university and his plague tract was widely circulating.^45^ After all, Jacmé was not mentioned in Jenštejn’s personal correspondence, which may suggest that he was added into the story after the fact. Jacmé’s renown was of key importance for the broader point that Jenštejn was trying to make throughout *De bono mortis*, namely that the power of communion was superior to even the most sophisticated forms of medicine. Jacmé served Jenštejn’s purpose because he represented the type of elite, learned physician who took healing into his own hands rather than acknowledging the power of the medicine of communion.

Jacmé and his colleagues were baffled by Jenštejn’s illness and they had no clue of how to treat him. Their astonishment only increased after Jenštejn was given an alternative treatment through which he was cured:

While I was lying there gravely sick and he [Jacmé] despaired of my life, he ordered that some pottage be given to me. He prohibited me from drinking wine altogether, except once or twice, doubly diluted, yet still I became increasingly ill. It happened one day that a certain student in the absence of others gave me as much food and drink as I liked. And at a time when I could barely eat two spoonfuls of pottage, I swallowed down eight glasses of strong wine and recovered to perfect health. Then after a little while the physician came to me, touched me with his hand and, truly amazed, began to shout, ‘He is healthy! He is healthy!’ Without doubt astonished, he began to inquire where my health had come from, and said to those who told him what had been done, ‘Alas for us! Often are they beguiled who strive to cure foreigners—who are evidently so different from us in life, customs and locale—with domestic medicines.’^46^

Jean Jacmé’s reaction to this healing is telling. Rather than acknowledge the power of the wine, Jacmé attributes the cure to the foreign complexion of Bohemians that differed from the Franks. Throughout this chapter of the text, Jenštejn implies that the wine is a symbol of communion. Although he does not explicitly state that the wine he drank in Montpellier was communion wine, he attributes his healing to Christ’s influence as the divine Physician. As Didier Lett has demonstrated in relation to the canonisation process of Nicholas of Tolentino, the failure of a physician in such a story did not necessarily reflect poorly on the practitioner. On the contrary, such stories indicated that the physician pushed his expertise to the limit and humbly yielded to the greater power and authority of the divine.^47^ In Jenštejn’s narrative, Jacmé is not presented quite so favourably. He asserts that the cure was due to the fact that the Bohemian complexion reacted to strange treatments that would not have been effective on domestic patients. Jenštejn too seems to frame much of his discussion of illness around complexion, but he attributes his healing to the wine and God’s influence. There is never any suggestion on Jenštejn’s part that the wine, which is implicitly representative of communion, would work only on his Bohemian complexion, in fact, he seems to suggest that Christ would heal all who placed their faith in him. Put simply, the two disagreed on the cure. For Jenštejn, the cure was due to Christ’s influence, whereas Jacmé deemed the cure to be effective only because of the strange complexion of Bohemian bodies that reacted to seemingly irrational remedies.

After recounting this incident, Jenštejn attempted to explain why physicians were not equipped to treat patients of different complexions. He argues that often a physician will treat all patients as if they share the same complexion as himself:

Of course, as it often happens, even certain physicians designate all other complexions according to their own. And, therefore, his medicines are to a greater extent known and through experimentation approved to share with all the rest of the patients.^48^

According to Jenštejn, the effectiveness of physicians’ medicines was measured through experimentation on local patients who likely shared the same complexion. Since physicians were focussed on those complexions that were familiar, they lacked the knowledge of how to treat those they encountered less often. Yet for Jenštejn, a master physician must gain knowledge and experience of treating various different types of patients. To make his point more emphatically, he draws an analogy between medicine and art:

For in the same way there is the example of the painter and sculptor of images, who preserve different singular properties in their art and images, shaping some oblong, others round, others thick and others slender. Thus, the work of any artist may be distinguished from students without the same skill of the masters. Hence, without doubt many illnesses and deaths are able to succeed on account of the natural impressions and qualities of physicians, to which they are more inclined.^49^

When confronted with the work of a master artist and a student, one can easily discern the difference. Where the skilled artist is able to depict numerous different shapes and forms in their work, it is implied that the student lacks the ability. Unskilled artists might be competent in producing one or two types of forms in their images, but they will not be able to recreate the multiplicity of forms found in the world. Medicine operates according to the same principals, namely, an unskilled student may only be able to treat one type of complexion or condition, whereas the master must have the skill, knowledge and experience to treat a multitude. Since some physicians are proficient only in treating the same ‘natural impressions and qualities’ that they themselves possess, patients with differing qualities suffer. The master physician does not suppose that all his patients will react in the same way to his treatments, but has a broad understanding of the diverse medicines that can be used to treat patients with varied complexions.

As we have seen, Jenštejn’s understanding of complexions was not dissimilar to those of physicians and medical authorities. Even in denouncing medicine Jenštejn invoked Galenic theory, as his illness and the troubles he faced in treatment derived from complexional difference. Yet Bohemians and Franks were not vastly different according to theories of complexion; as we have seen, Jenštejn fared much better in Paris, where the more moderate heat suited him. Indeed, medieval notions of complexional difference were not limited to peoples of vastly distant lands, but could be more subtle. The differences may not have been immediately apparent to practitioners and patients, yet the distinction had profound consequences for illnesses and treatments. Just as Jenštejn’s body struggled to deal with the difference in climate, so Jean Jacmé and others failed to treat those with unfamiliar complexions. Clearly, for Jenštejn, even elite physicians were underequipped to treat a diversity of patients, but Christ could step in with his salvific medicine of communion.

## Criticisms of the Theory and Practice of *medicus carnalis*

Jan of Jenštejn accepts that his Bohemian complexion may not have been suited to the Mediterranean heat, but this does not mean that he finds communion to be an effective remedy only to those of his complexional disposition. On the contrary, he laments that more do not take communion frequently to heal spiritual as well as physical ailments. Jenštejn takes a harsh line in condemning what he terms ‘medicus carnalis’, namely the physician of the body, for failing to acknowledge the healing power of the sacrament of the altar or that spiritual deficiencies could be the cause of physical illnesses. In *De bono mortis*, he laments:

A physician of the body therefore labours in vain when he heals the body and he is ignorant of the plight of the soul whence sickness springs, and therefore there is no doubt that physicians are deceived and may deceive others on that account. For the physician of the body deals in diseases or feebleness, which he perceives by sight and touch and other palpable experiments; insofar as he has the power to cure by that which is palpable, he depends on medicines. But those things which are invisible and intangible are not discerned by the outward senses. Since spirits and souls [are] internal and invisible passions, no one can comprehend by reason and similarly cure them. For as it is written in the Book of Wisdom [16: 12], those who were infected by diverse sicknesses were cured not through that which they saw, but were cured through the Saviour of all: ‘neither herb nor poultice healed them, but the Word of the Lord that heals all.’ For that reason I say this, because even with visible disease often the eyes and hands of physicians are deceived.^50^

For Jenštejn, it is all very well attending to the humours and providing patients with medicines and diets, but if the soul is unwell, then these treatments will come up short. Jenštejn draws on Wisdom 16, where the Egyptians were bitten and stung by the very animals they worshiped. This passage provides an apt analogy because he presents physicians as causing the illnesses that they looked to cure. Like the Egyptians of Wisdom 16, physicians trust in something other than God, namely medical authorities, and in turn cause more infirmities than they cure. By extension, those patients who trust physicians over God’s will and the power of the sacraments face the prospect of increased illness.^51^

Central to Jenštejn’s criticism of earthly physicians of the body were the ways in which physicians interpreted the works of their authorities. His criticism was part of a larger trend that emerged in the second half of the thirteenth century. Sceptics suggested that medical masters were overly concerned with theory and rigidly stuck to their authorities instead of adapting to patients’ needs.^52^ Jenštejn quotes the opening lines of Hippocrates’s *Aphorisms* to propose that medical practitioners have little right to call themselves experts when they lack practical experience:

Behold how doubtful and uncertain is the profession of medicine, and how untried the science of medicine and of physicians. Accordingly, they call the experts ‘physicians’, and Hippocrates is quoted, who said: ‘Life is short, but art is long, experience is false, judgement difficult.’^53^ And as if to conclude I will add that the learned and proven physician ought to have experience and knowledge of all physicians who have gone before; and thus he will hardly flee those in the multitude whom he daily cures and visits, because he should not offend in anything and should not incur irregularity. Because he who offends in one thing is guilty of all things, and thus he gravely sins.^54^

Jenštejn’s scepticism is not directed towards Hippocrates himself—indeed he holds a degree of reverence for the father of medicine. Rather Jenštejn argues that physicians neglect their most basic duties of visiting their patients and thus they fail to gain experience. By failing in this one area, they let down the entire profession, especially as there seemed to be a lack of clear punishments for negligent physicians.^55^

Although Jenštejn did not directly attack medical authorities, he pointed out their shortcomings. He contends that not everything written by medical authorities should be accepted without scrutiny. After all, they lived long ago, in regions with vastly different living conditions to physicians of his present:

Let us not judge harshly Hippocrates, Galen, Avicenna and other physicians far away in remote regions, where the elements, herbs, conditions of men and other circumstances may behave differently or even contrarily. In some lands too much abstinence is practised. In others, an excessive abundance flows out of people. And yet, they are found to be more robust and live longer than [those who practice] abstinence from overindulgence, when, according to the dictates of reason, the opposite ought to be preferable. Therefore, since they [medical authorities] had not been everywhere and did not subject their own eyes to all things and other circumstances, why in their codices do they teach and presume to cure the weaknesses, infirmities and diseases of the whole world?^56^

Jenštejn makes a valid point because, as we have seen from his illness in Montpellier, geographical factors were especially important to medicine. Hippocrates, Galen and Avicenna were exposed to different complexions and body types and so could not possibly speak to the types of complexions of people in late-medieval Europe. On a more practical level, medical authorities such as Hippocrates, Galen and Avicenna had access to different ingredients for medicines than a physician such as Jean Jacmé in fourteenth-­century Montpellier. Of course, many ingredients remained the same throughout the centuries, but this is not the point of Jenštejn’s argument, which lacks nuance in this area. Jenštejn maintained that the time that had passed since the fathers of medicine lived rendered their writings inadequate, as different illnesses and ways of life had developed. Considering this, why would physicians continue to refer to them?

It was not simply that place and time were different for medieval physicians, but also circumstance. Jenštejn considered himself to live at the end of ages, a time in which spiritual medicines were desperately needed to heal humanity before the imminent apocalypse. In *De bono mortis*, Jenštejn contributes to an apocalyptic trend in Bohemia that was fostered by the likes of Jan Milíč of Kroměříž, Matěj of Janov and Mateusz (Matthew) of Kraków (d. 1410), and exacerbated by the Great Western Schism.^57^ Jenštejn blends this apocalyptic theology with a medical discourse in which physicians needed to focus on spiritual medicines above the medical traditions of old. He explains:

We have now reached the end of ages […], in the present our land has turned *into barrenness* overflowing with *the wickedness of those that dwell therein* [Ps. 106: 34], in which we now see that the virtues of herbs, trees, fruits, spices, grains and the various liquids that come from them are so ineffective that they can have little or no effect on health. Whence a sound and agreeable argument of reason can be made of the old traditions of medicine which benefited in ancient times; in any case, little or no effect can be made for the sake of health on account of the ancient conditions [of the authorities], a lack of a greater number of creatures and on account of the End Times, where all await the final end of all and require other medicines.^58^

As was typical for apocalyptic discourses, Jenštejn contrasts the ancient world with the moribund present.^59^ The recipes that were used by medical authorities were undoubtedly successful in curing maladies, but the ingredients to which they had access were more abundant and potent in their own time. In his present day, Jenštejn presents a pessimistic view of a decayed state of the world in which the same ingredients are either ineffective, scarce or extinct.

Since Jenštejn explains that the medicines of old would not work in his present, he argues that adaptation is needed. Unfortunately, Jenštejn claims, physicians are unwilling to deviate from their outdated authorities. He was not the only one to make this criticism and in fact contributed to a broad trend of scepticism towards university medical education. The French master surgeon Henri de Mondeville (d. 1320), presented analogous thinking when he compared ancient medical authorities to a decrepit old dog whose owner did not have the heart to replace him with a younger animal. Henri also compared medical authorities with ancient Greek architects whose work was once admired but had to be demolished to make way for newer buildings.^60^ Jenštejn makes the same point with a series of different analogies:

according to some, at various times it is permissible to change statutes and to establish new laws on account of recent crimes, new punishments on account of diverse contingencies, diverse remedies on account of the plurality of illnesses; now in the most recent age, having considered the circumstances, new medicines are poured forth, which in the medical tradition of the prudent are legitimately considered by industrious physicians. To saving grace, I say thus: it is expedient of physicians to emend the ancient codices in certain places, correct or even abolish them altogether. And having considered the infirmity of the human condition etc., which in recent times possess all to a greater extent, it is more important for man to invent new medicines. As it is written: *You will throw away the old with the new arriving* [Lev. 26: 10]. And it is not found in their canon to compose new combinations of herbal powers and various pigments in order to adapt with time and human frailty. But if otherwise [they throw out the new for the old] they cause them to err; he who is wise does not know it at all. When most consider that they are rebuffed and by the favour of physicians scorned, the infirm are sometimes treated by the industry of laymen or wicked women with new and unusual herbs and medicines.^61^

Clearly, in the End Times new medicines were required to heal spiritual wounds and prepare for the coming Final Judgement. In aid of this, the codices containing the works of medical authorities had to be altered or else cast aside. Yet with physicians sticking so rigidly to their authorities and ignoring any innovations, the patient is forced to search elsewhere for treatment. If the physician rejected the patient’s plea for new remedies, the patient had little choice but to turn to the uneducated. It is implicit within this quotation that turning to the industry of the uneducated laymen or wicked women was a risk. After all, these untrained empirics used medicines, charms and amulets that had no theoretical underpinning but were proven by experiment alone.^62^ Jenštejn treats these types of alternative healers who defied learned medical theory with just as much scorn as elite physicians. While Jenštejn acknowledges that there were certain ‘industrious physicians’, most, whether university-trained or not, were flawed in comparison to the care offered by Christ. Placing one’s trust in any earthly healer was hence misguided if the healer was not prepared to consider new medicines to remedy the increasingly corrupt state of humanity in the End Times.

Jenštejn clearly had a decent grasp of medical theory, yet his argument should not be taken to represent the ways that medieval physicians adapted received medical knowledge. First, physicians are known to have altered recipes and remedies for their specific needs. Irma Taavitsainen has shown that the genre of the recipe book was more ‘adaptable and flexible’ than typical academic works, and scribes and owners modified recipes instead of rigidly following an authoritative exemplar.^63^ Physicians did not employ the same medicines across the known world, but often adapted recipes to their circumstances and needs. Recipes were updated or altered and authors would also recommend alternative ingredients for those with limited access to resources.^64^ Second, medical authors did not simply rely on ancient works for practical medicine in the later Middle Ages, as a large selection of medieval works and recipes became widely popular across Europe.^65^ Undoubtedly the works of Hippocrates, Galen and Avicenna continued to be staples of the physician’s theoretical education, but physicians turned to other, more recent works for recipes and treatments. Therefore, while Jenštejn demonstrated a relatively sophisticated knowledge of the different complexions of patients and the problems this could pose to healers, he failed to understand the nuances with which physicians used their authorities. This oversight could arise from the fact that he was not a practitioner, or perhaps his argument reflected a purposeful manipulation to suit his rhetorical needs. Whatever the case may be, Jenštejn presented a clear argument as to why earthly healers were so unsuccessful: when they were not consulting ancient, outdated tomes, they were ignoring the ways in which illness could be a direct result of a spiritual sickness, especially in the End Times.

## Jan of Jenštejn in Nuremberg and the Superiority of *Christus Medicus*

In pointing out the negligence of physicians, Jenštejn sought to expose a lack of familiarity with what was required to heal spiritual illnesses. Christ, by contrast, is intimately acquainted with the soul and its healing. Acting as both a physician and patient, Christ is the Man of Sorrows who turned the great suffering of his Passion into an act of healing through his grace. This ultimate sacrificial act of healing was re-enacted regularly through the sacrament of the altar, where Christ became both the physician and medicine:

In fact he is a health-giving holy Physician for us and a wholesome medicine, for he has known our form for eternity, and indeed we do not require another who is sufficient to our health, [Isaiah 53: 3] ‘as a man of sorrows and one who knows weakness.’^66^

Through the Passion, Christ took on the sins of the world and became acquainted with every type of pain or infirmity that could afflict the soul. Christ’s Passion made him the perfect physician because his first-hand experience of suffering enabled him to empathise with patients. In fact, this compassion and empathy was seen as a vital part of healing and convalescence.^67^ Christ’s compassion and expertise presents a stark contrast to elite physicians such as Jean Jacmé, who seemingly had little empathy for Jenštejn’s illness in Montpellier or much of a clue of how to treat him.

Jenštejn does not leave his claims about Christ’s healing power to stand without proof; he turns to a personal example as a *probatum est*. Jenštejn recalls that he sat down to dinner with a number of guests, including Mikuláš (Nicolas) Biceps of Jevíčko (d. 1390/1), a Dominican master at Prague who was an opponent of frequent communion at the university. Following the dinner, all of the guests contracted the plague.^68^ Although Jenštejn describes Biceps as young and strong, he died after two days and nights, while the other guests recovered because, unlike Biceps, they took communion each day until they were healthy.^69^ It is no coincidence that Biceps, with his stance against frequent communion, was the only guest to succumb to the illness; his death served Jenštejn’s rhetorical purpose.^70^ What does it say about the power of frequent communion when Jenštejn, by all accounts a sickly figure, survived the plague and a strong, young man such as Biceps died? It is significant that Jenštejn grounds the practice of frequent communion in a medical discourse that is not just directed towards spiritual but also bodily health. For Jenštejn, the sacrament of the altar was a powerful remedy that could function as a medicine for the most serious bodily infirmities.

Yet it was not simply the sacrament of the altar that was required to heal a suffering patient; a devotional attitude was paramount. From March to the end of September 1380, Jenštejn accompanied King Wenceslaus IV as his chancellor in German lands.^71^ Around April, Jenštejn fell ill in Cologne. He reported in a letter to Jan of Středa, bishop of Olomouc (d. December 1380), that he was injured in his body and limbs to such an extent that physicians feared for his life. Jenštejn rejected the medicine that was prescribed by his physicians and by the grace of God he recovered enough strength to continue his journey. He reports to Jan of Středa that, having gained some strength and seeking to avoid the plague that was on the rise in Bohemia, he and King Wenceslaus would travel on from Cologne to Nuremburg.^72^

It was in Nuremberg that Jenštejn suffered his most severe illness of the trip. This malady began with a fever that made him feel alternately hot and cold. Then he was confined to his bed for two months and subsequently lost a great deal of weight.^73^ In a letter sent to Archdeacon Přibyslav of Horšovský Týn (in which Jenštejn invokes the *pluralis majestatis* because he was now archbishop of Prague), Jenštejn provides the greatest detail on his symptoms:

for eight whole weeks a fever agitated us excessively and a twofold affliction exhausted us, that is to say, a double tertian fever that we had every day, which now excessive cold wickedly disturbed our body so that the cold shook us with a strong shivering, then it was scorching with a very hot fire so that we thought we had been consumed by ravenous fires; indeed because we were exhausted of energy, deprived of our usual strength and emaciated, our nerves displayed their number to those who looked on.^74^

Petr Klarifikátor describes this same incident, explaining that when physicians doubted his recovery, Jenštejn began to lament his wasted youth.^75^ Jenštejn invoked the protection of Christ and Mary, promising that after his recovery he would vow to live an ascetic life. He was miraculously cured on a Saturday, the day attributed to the Blessed Virgin:

But still, with the glorious protection of the Mother, whose day it was then, aided by the merciful Almighty, who after the rod of correction extends the staff of his merciful pity, with the fever released, the body took up again the fire of natural heat and, strengthening our arms stronger day by day, the empty skin seems to take up a mass of flesh, the nerves too recover; by the strength of the vital spirits, they hide under the thickness of the flesh, so that nothing but health remains from the gift of God.^76^

After his recovery, Petr Klarifikátor reports that Jenštejn eventually returned to Bohemia and increasingly spent his time at the monastery of Roudnice, which he supported throughout the rest of his life.^77^ He even resided there after he was removed from his post as archbishop of Prague.^78^ According to Klarifikátor, this bout of illness and the subsequent examples we have already explored enabled Jenštejn to attain an almost saintly status among the inhabitants of Roudnice.^79^ Indeed, Klarifikátor follows the common archetype of the suffering saint in his work, attempting to push the image of Jenštejn as a divinely favoured figure.^80^ This miracle also had a political component; in 1386, Jenštejn and his milieu began a campaign for the Feast of the Visitation of the Blessed Virgin Mary to be accepted as an officially sanctioned feast day, first in his own archdiocese and then later throughout the Church.^81^ The experience certainly convinced Jenštejn that the feast was suited to the needs of the Church, which was suffering because of the Great Western Schism. Further, the healing of Jenštejn demonstrated Mary’s favour for the Archbishop, which would be proven time and again through numerous examples of illness and recovery across his life.

Nevertheless, it appears that Petr Klarifikátor and Jenštejn had different ideas concerning the effectiveness of medicine, which might be explained by the differing genres of their works. For Klarifikátor, who explicitly sought to write a hagiographical text, the treatment of physicians was useful alongside the healing of Christ because the divine Physician used earthly practitioners as assistants in healing spiritually ill. For Jenštejn, whose text featured numerous sceptical reproofs of medicine as part of his theology, earthly practitioners were entirely ineffective and were nothing but a hinderance to the work of the divine Physician. The different accounts of Jenštejn’s deadly fever in Nuremberg most clearly illustrate this difference of opinion. While Klarifikátor acknowledged that Jenštejn was treated by ‘stupentibus medicis’ together with divine intervention, Jenštejn claimed that he was healed by Mary and Christ alone and that the physicians who attempted to cure him were incompetent. In fact, throughout *De bono mortis* physicians consistently seemed to worsen Jenštejn’s state of health.

Possibly the most striking denunciation of medicine by Jenštejn comes in the example with which we started—in which Jenštejn fell into a severe illness three miles outside of Prague on his return from Rome. After Jenštejn was cured by the Eucharist, he explained that his physicians were still concerned for his health and recommended various conservative and convalescent medicines:

for after I had taken the saving Physician and salubrious medicine in Christ’s body, physicians of the body [*carnales medici*] pressed with rude warnings that I should accept bodily medicine. I said to them that I would not take anything other than that which heals me. For I thought in my mind that it would not be expedient for me to take any further medicines if I wanted to be perfectly cured; for if I did otherwise, I know for certain that I would relapse into my former illness. But with the physician persisting and insisting along with others that I should take something for my maintenance, I, wretched and inclined to frailty, have been subject to unpleasantness so that I may become obedient and not spurn the physicians, who err in worrying about my health; I have received bodily comfort and to a degree have lost spiritual comfort due to the well-being of the body.^82^

Although Jenštejn gave in to the badgering of the physicians and agreed to take some medicine for his bodily comfort, he was aware of the risks to his spiritual health. For Jenštejn, this episode highlighted the short-sightedness of bodily physicians because they exchanged spiritual health, which is eternal, for temporary physical comfort. After all, throughout his life Jenštejn became quite familiar with bodily discomfort and even celebrated his illnesses as tools for penitential development.

Despite Jenštejn’s insistence that earthly medical practitioners were impotent, he acknowledges that there had been various physicians in the past who ought to be revered:

One may note Luke the physician, whose renown is in the Gospel, and the glorious martyrs Cosmas and Damian, blessed Panthaleon, too, with the other saints who were supported on both sides by divine grace alone: they came forth as physicians of both body and soul, although more of the soul than the body.^83^

Luke the Evangelist was of course an entirely trustworthy physician because he was an apostle and the Holy Spirit worked through him. Cosmas and Damian are the saints most regularly associated with physicians, surgeons and pharmacists. They were physicians who would not accept money for their treatments and were hence dubbed the ‘silverless’ saints. St Panthaleon became more frequently invoked as a patron saint of physicians and midwives after the first outbreak of the plague in 1348–50. He shared a connection with Cosmas and Damian because in the Eastern Church, Panthaleon was considered to be another of the silverless saints.^84^ The stereotype of the avaricious physician was common throughout late-medieval European texts, and hence it is not exceptional to see such praise for physicians who treated patients charitably.^85^ Nonetheless, Jenštejn did not infer that earthly practitioners sought only to make a profit, as many critics and satirists suggested. Jenštejn seemed to be uninterested in whether physicians charged too much for their services or not. Instead Jenštejn invoked the silverless saints because of their dedication to healing the soul as well as the body. In effect, these physicians imitated Christ in their spiritual practice and dedication to treating sin in a penitential manner.

Given the high standard to which Jan of Jenštejn held physicians, it is hardly surprising to deduce that *De bono mortis* shows the field of medicine to be flawed. It is telling that the only physicians he praises are saints and Christ the divine Physician. If we are to take Jenštejn at his word, we are left to assume that all earthly physicians are likely to be ineffective. For Jenštejn, healing was provided by Christ through devotion and communion, which was proven time and again through his personal anecdotes of travel and illness. In his autobiographical narratives, Jenštejn paired knowledge and experience, but not in the same manner described in Hippocrates’s *Aphorisms*. Instead of studying medical authorities and precedent cases, Jenštejn asserted that knowledge of Christ and experiencing him through frequent communion was central to spiritual as well as physical health. Of course, the text is purposefully exaggerated and Jenštejn does not truly reject medicine. Despite his insistence on the flawed nature of medicine, he describes his illnesses in relation to Galenic theory and invokes medical authorities at various points throughout his *De bono mortis*. In other words, Jenštejn engages with concepts of health through the very terms, concepts and theories of academic medical discourse that he derides.

Still, Jenštejn’s narratives are not simply religious exempla used to make a polemical point about frequent communion; his autobiographical writings have value for the study of the history of medicine. As his trips to Rome, Montpellier, Cologne and Nuremberg illustrate, travelling as a sickly person in the Middle Ages was daunting. Not only did travelling patients have the climate and its effects on their complexion to worry about, but they also had to contend with the mixed quality of care provided by unfamiliar healers. Jenštejn therefore raises pressing questions for travelling patients. Was a physician equipped to deal with different body types and complexions? Would the physician rely on seemingly outdated authorities? Was a physician equipped to treat the soul as well as the body? Thus, whether Jenštejn gives us an accurate impression of his illnesses and travels or not, his work provides a means of conceptualising the problems that itinerant patients may have faced across Europe.

## Footnotes

1 For Jenštejn’s involvement in the establishment of the feast, see Ruben E. Weltsch, *Archbishop John of Jenstein, 1348–1400: Papalism, Humanism and Reform in Pre-Hussite Prague* (The Hague: Mouton, 1968), 88–91.

2 Jan Sedlák provides an edition of this extract in his *M. Jan Hus* (Prague: Dědictvi s. Prokopa, 1915), 45*–46*: ‘a Pragensi urbe miliaribus tribus tantum distarem, repentinus in me languor irruit horribili febre et naturalis unius diei spacio miserabiliter me quaciendo detinuit et ad usque internecionem prostravit. Vehementissimo igitur dolore et gravi infirmitate per plures dies consumpto medicis de vita desperantibus et alii spe frustrate similiter fuerunt noti videlicet mei amici et proximi, ego quoque similiter non me amplius vivere debere nullatenus reputabam […]. Accumbebam ergo ego flexis poblicibus tunc quodam cervicali, ast debilitate me nimia invadente amencia quedam succrescebat, quam cum percepissem notabiliter augeri, cepi surgere et ad presbiterum festinare […]. Presbitero autem retardante in salutaris hostie tradicione, *contexerunt me tenebre* [Psalms 54: 6] et apertis oculis videre non poteram, obsurdui et obmutui penitus et prout astantes asseruerunt et hii qui me tenebant plurimi ossa mea ad instar mortuorum collidi et frangi videbantur, ac signa morituri hominis notabiliter foris aliis apparebant, intus vero anima mea inenarrabili dolore torquebatur.’ The text survives in three manuscripts: Biblioteca Apostolica Vaticana, Vatican Lat. 1122, ff. 65^v^–86^r^ <https://opac.vatlib.it/mss/detail/10990>, accessed April 09, 2024; Prague, Národní knihovna (PNK), Osek 37, ff. 130^r^–150^v^ <https://www.manuscriptorium.com/apps/index.php?direct=record&pid=AIPDIG-NKCR__OSEK_37_____0D6W3OF-cs>, accessed April 09, 2024; finally, the text survives with one missing folio (between folios 12 and 13) in Wrocław, Biblioteka uniwersytecka, I F 777, ff. 1^r^–25^v^ <https://www.bibliotekacyfrowa.pl/publication/32518>, accessed April 09, 2024. Throughout this article, where possible I use Sedlák’s edition and elsewhere my transcriptions come directly from the manuscripts.

3 Jaroslav V. Polc, *Česká církev v dějinách* (Prague: Akropolis, 1999), 304–5.

4 Helena Krmíčková, ‘Petri Clarificatoris Vita domini Iohannis, Pragensis archiepiscopi tercii (Peter Clarificators Vita domini Iohannis, Pragensis archiepiscopi tercii)’ in *Querite primum regnum Dei. Sborník příspěvků k poctě J. Nechutové*, ed. Helena Krmíčková (Brno: Matice moravská, 2006), 453; Jaroslav Kadlec, ‘Petr Klarifikátor, duchovní vůdce a životopisec arcibiskupa Jana z Jenštejna’, *Sborník Katolické teologické fakulty*, 1998, 1, 101–50.

5 Jenni Kuuliala, *Saints, Infirmity and Community in the Late Middle Ages* (Amsterdam: Amsterdam University Press, 2020), 11–14, 32–40. For more on the common topoi of suffering in the lives of pious figures, see Esther Cohen, *The Modulated Scream: Pain in Late Medieval Culture* (Chicago: University of Chicago Press, 2010), 4–8; Irina Metzler, *Disability in Medieval Europe: Thinking about Physical Impairment during the High Middle Ages, c. 1100-1400* (London: Routledge, 2006), 134–138.

6 Louise Elizabeth Wilson, ‘Miracle and Medicine: Conceptions of Medical Knowledge and Practice in Thirteenth-Century Miracle Accounts’, in Anne Kirkham and Cordelia Warr, eds, *Wounds in the Middle Ages* (London: Routledge, 2014), 63–86; Didier Lett, ‘*Judicium Medicine* and *Judicium Sanctitatis*: Medical Doctors in the Canonization Process of Nicholas of Tolentino (1325): Experts Subject to the Inquisitorial Logic’, in Kirsi Salonen and Sari Katajala-Peltomaa, eds, *Church and Belief in the Middle Ages: Popes, Saints, and Crusaders* (Amsterdam: Amsterdam University Press, 2016), 154–156; Metzler, *Disability in Medieval Europe*, 143–150.

7 Sedlák, *M. Jan Hus*, 47*: ‘Contingit interea michi in memoriam revocari, qualiter adhuc communicare debeam; nam in permissis pressuris oblitus eram de communione. Dixi ergo mecum: Etsi oculi mei caligant [cf. Genesis 27: 1], ut intueri dominum meum non valeam, saltem os meum aperiam, ut hoc venerabile recipiam sacramentum. Sic sicque os meum aperui et ad reverenter suscipiendum corpus domni *attraxi spiritum* [Psalms 118: 131]. Et statim aperti sunt oculi mei et presbiterum ante altare exspectantem cum eukaristia cum ceteris circumstantibus illitum lacrimis vidi. Cumque panem vivum, qui *dat vitam mundo* [John 6: 33; 41], conspexi resolutum est eciam vinculum labii et dicere cepi: “Domine mi rex [1 Kings 24: 9].” Sacerdos autem mox michi corpus dominicum porrexit et statim a me omnis dolour abscessit modico vestigio debilitates remanente.’ Gottfried Vielhaber, ‘Der *Libellus de bono mortis* des Erzbischofs Johann von Jenstein’, *Festschrift des Vereines für Geschichte der Deutschen in Böhmen, seinen Mitgliedern gewidmet zur Feier des 40jährigen Bestandes* (Prague: Verein für Geschichte der Deutschen in Böhmen, 1902), 164.

8 There is not space in the present article to discuss all the nuances of this discourse of illness, which has already been treated at length. In particular, see Metzler, *Disability in Medieval Europe*, 47–48; Cohen, *Modulated Scream*, 187–9.

9 Vielhaber, ‘Der *Libellus de bono mortis* des Erzbischofs Johann von Jenstein’, 163.

10 See David R. Holeton, *La Communion des tout-petits enfants: Étude du mouvement eucharistique en Bohème vers la fin du Moyen-Âge*, Bibliotheca « Ephemerides Liturgicae » Subsidia, vol 50 (Rome: C.L.V.-Edizioni Liturgiche, 1989); idem, ‘The Bohemian Eucharistic Movement in its European Context’, *Bohemian Reformation and Religious Practice*, 1996, 1, 23–48.

11 Jaroslav V. Polc and Zdeňka Hledíková*, Pražské synody a koncily předhusitské doby* (Prague: Karolinum, 2002), 243–45; Ivan Hlaváček, ‘Zur böhmischen Inquisition und Häresiebekämpfung um das Jahr 1400’, in *Häresie und vorzeitige Reformation im Spätmittelalter*, ed. František Šmahel, Schriften des Historischen Kollegs, vol 39 (Munich: De Gruyter, 1998), 116–117.

12 Rostislav Nový, ‘Ženské řeholní a laické komunity v předhusitské Praze’, *Documenta Pragensia*, 1996, 13, 41–46.

13 Polc, ‘Statutes of the Synods of Prague (1386–1395)’, 445–447: ‘veri penitentes, cum sint tamen confessi et contriti et digni, possint communicare quotiescumque affectant.’ For the decrees, see: František Šmahel, *Husitská revoluce, 2, Kořeny české reformace* (Prague: Karolinum, 1996), p. 207.

14 Weltsch, *Archbishop John of Jenstein*, 101. For more on St Ambrose and his treatment of the death of sinners and the body, see *S. Ambrosii* De bono mortis: *A Revised Text with an Introduction, Translation and Commentary*, ed. and trans. William Theodore Wiesner (Washington: Catholic University of America Press, 1970), especially pp. 18–23, 29–30. Nicolette Zeeman records the same types of narratives in John Bromyard’s works. *The Arts of Disruption: Allegory and Piers Plowman* (Oxford: Oxford University Press, 2020), 302–303. See also Mark Chinca, *Meditating Death in Medieval and Early Modern Devotional Writing: From Bonaventure to Luther* (Oxford: Oxford University Press, 2020), 1–9.

15 See Nancy Siraisi, *Medieval and Early Renaissance Medicine* (Chicago: University of Chicago Press, 1990), 39–42, 153, 166; Katherine Park, ‘Medicine and Society in Medieval Europe 500–1500’, in *Medicine in Society: Historical Essays*, ed. Andrew Wear (Cambridge: Cambridge University Press, 1992), 59–90, especially pp. 73–75; Simone C. Macdougall, ‘The Surgeon and the Saints: Henri de Mondeville on Divine Healing’, *Journal of Medieval History* 26: 3 (2000), 254–56; Joseph Ziegler, ‘Practitioners and Saints: Medical Men in Canonization Processes in the Thirteenth to Fifteenth Centuries’, *Social History of Medicine*, 1999, 12, 191–225; Ronald C. Finucane, *Miracles and Pilgrims: Popular Beliefs in Medieval England* (London: J.M. Dent, 1995), 60–99; Iona McCleery, ‘Christ More Powerful than Galen? The Relationship between Medicine and Miracles’, in Matthew M. Mesley and Louise E. Wilson, eds, *Contextualizing Miracles in the Christian West, 1100–1500: New Historical Approaches*, Medium Ævum Monographs, vol 32 (Oxford: Society for the Study of Medieval Languages and Literature, 2014), 127–54, especially p. 131. Many of these same elements are commonly found in the *narrationes* of *chirurgiae*, that is, books of surgical instruction, a genre of texts produced and circulated in large numbers in the later Middle Ages. Peter Murray Jones, ‘The Surgeon as Story-Teller’, *Poetica*, 2009, 72, 80.

16 Vielhaber, ‘Der *Libellus de bono mortis* des Erzbischofs Johann von Jenstein’, 163. Similarly, Pavlína Rychterová in ‘Charisma und charismatische’, 347–51 identifies a theme of ‘exklusive Frömmigkeit’ that runs throughout *De bono mortis*. Focusing on a different but connected aspect of the text, Marie Bláhová notes that the work is an early example of a growing trend of autobiographies in the later Middle Ages. ‘Autobiografie v českém středověku’, *Historická sociologie*, 2016, 8, 51–74.

17 Pavlína Rychterová, ‘Charisma und charismatische Legitimation in der Vita von Johannes von Jenstein’, in Jiří Fajt and Andrea Langer, eds, *Kunst als Herrschaftsinstrument: Böhmen und das Heilige Römische Reich unter den Luxemburgern im europäischen Kontext* (Berlin: Deutscher Kunstverlag, 2009), 347–351.

18 Weltsch, *Archbishop John of Jenstein*, 100.

19 Peter Murray Jones, for instance, demonstrates the lasting impact of Roger Bacon’s condemnation of scholastic medicine, especially in his *De erroribus medicorum*. Bacon’s criticisms had a long legacy among the Mendicant orders across Europe, which showed a preference for practical medicine and pharmacology in many of their surviving medical compilations. *The Medicine of the Friars in Medieval England*, Health and Healing in the Middle Ages, vol 5 (York: York Medieval Press, 2024), 100–102, 136–39. See also Chiara Crisciani, ‘Per una ricerca su “experimentum”-“experimenta”: riflessione epistemologica e tradizione medica (secoli XIII–XV)’, in Pietro Janni and Innocenzo Mazzini, eds, *Presenza del lessico Greco e latino nelle lingue contemporanee* (Macerata: Università degli Studi, 1990), 9–49; *Expertus sum: l’expérience par les sens dans la philosophie naturelle médiéval: actes du colloque international de Pont-à-Mousson, 5–7 février 2009*, ed. Thomas Bénatouïl and Isabelle Draelants (Florence: SISMEL edizioni del Galluzzo, 2011).

20 See Michael R. McVaugh, ‘The Nature and Limits of Medical Certitude at Early Fourteenth-Century Montpellier’, *Osiris*, 1990, 6, 62–84; Mark G. Jordan, ‘The Disappearance of Galen in Thirteenth-Century Philosophy and Theology’, in *Mensch und Natur im Mittelalter*, by Albert Zimmerman and Andreas Speer (Berlin: de Gruyter, 1992), 703–13; Danielle Jacquart, ‘Theory, Everyday Practice and Three Fifteenth-Century Physicians’, *Osiris* 6 (1990), 140–60.

21 The letters are edited in Johann Loserth, ‘Beiträge zur Geschichte der husitischen Bewegung, I. Der Codex epistolaris des Erzbischofs von Prag Johann von Jenzenstein’, *Archiv für österreichische Geschichte*, 1877, 55, 267–400. On pp. 294–298, Loserth provides a table that indicates where each letter was sent from, which also serves as an overview of Jenštejn’s travels.

22 Zdeňka Hledíková, ‘Lastnoročne opombe in korekture Jana z Jenštejna v njegovem epistolariju’, *Revija za umetnost in humanistiko / Ars and Humanitas*, 2014, 8, 72–86.

23 Of course, there was nothing inherently misleading about this approach to biography, as hyperbole was an established literary convention in such devotional works. David Bates, Julia Crick and Sarah Hamilton provide a comprehensive introduction to this matter and biography more broadly in the Middle Ages in their ‘Introduction’, in *Writing Medieval Biography, 750–1250: Essays in Honour of Frank Barlow*, edited by David Bates, Julia Crick and Sarah Hamilton (Woodbridge: Boydell & Brewer, 2006), 1–14; for the Bohemian context, see Bláhová ‘Autobiografie v českém středověku’, 52–56. Jenštejn’s letters and *De bono mortis* can also be classified as what Esther Cohen labels ‘alter-documents’, or autobiographical works that describe sensations of pain, illness and injury. Cohen, *Modulated Scream*, 17.

24 McCleery, ‘Christ More Powerful than Galen?’, 131.

25 Iona McCleery, ‘Saintly Physician, Diabolical Doctor, Medieval Saint: Exploring the Reputation of Gil de Santarém in Medieval and Renaissance Portugal’, *Portuguese Studies* 21 (2005), 113–114, 121.

26 Andrew Crislip, *Thorns in the Flesh: Illness and Sanctity in Late Ancient Christianity* (Philadelphia: University of Pennsylvania Press, 2013), 1–6; Kuuliala, *Saints, Infirmity and Community*, 11–14.

27 Wilson, ‘Miracle and Medicine’, 68–73.

28 . Krmíčková, ed, *Vita domini Iohannis*, 453.

29 Jan of Jenštejn studied in Paris, Montpellier, Padua and Bologna between 1370 and 1376, and associated with some eminent French masters. Weltsch, *Archbishop John of Jenstein*, 11–12.

30 Loserth, ‘Beiträge zur Geschichte der husitischen Bewegung’, epistulum 64, 384–85: ‘Nam inter cetera calores vivificos suo fervore Phebus in tantum propinat, quod humore interius dilatato sudores foris largiflue propellantur, sic quod faciem crebro sudore perfusam frequenter manu detergam.’

31 Loserth, ‘Beiträge zur Geschichte der husitischen Bewegung’, epistulum 64, 385: ‘Hiis itaque molitis nescio quid faciam, nec siti consulere, nec calorem eicere, nec egrotantis burse valeo vires repetere, et sic undique circumseptus instanciis quasi in laqueo positus nescio quid eligere et in quibus constanciam mentis indubitanter valeam consulere; et sic ex his multa mala resultant, nam mens vaga girat in desideriis plurimum occupata, tediumque corpus conmacerat, ita ut nec studio possim iuste intendere nec quasi in plura divisus parti cui debeam adherere.’

32 Polc, *Česká církev v dějinách*, 299.

33 Wilson, ‘Miracle and Medicine’, 69; Iona McCleery, ‘*Multos ex Medicinae Arte Curaverat, Multos Verbo et Oratione*: Curing in Medieval Portuguese Saints’ Lives’, in *Signs, Wonders, Miracles: Representations of Divine Power in the Life of the Church*, ed. Kate Cooper and Jeremy Gregory, Studies in Church History, vol 41 (Woodbridge: Boydell, 2005), 196.

34 Yet Jenštejn did not always suffer from the climate in Montpellier. One more letter sent from Montpellier to Beneš of Hořovice survives. The letter appears on folio f. 46^r^, earlier in the manuscript than the other letter sent from Montpellier on folio f. 48^v^, perhaps suggesting that it was written before Jenštejn’s complaint about the heat, yet the content matches the same themes of illness from the later correspondence. Jenštejn declares: ‘Nulla alia pro nunc michi occurrunt, nisi quod dei summa operante virtute sanus sum ac hilaris, prout terrena motibus supernorum reguntur siderum’ [Nothing else occurs to me for the present, except that by the supreme working power of God I am healthy and cheerful, as the earthly motions are governed by the heavenly motions of the stars]. Loserth, ‘Beiträge zur Geschichte der husitischen Bewegung’, epistulum 57, 381. In other words, even if intense heat disturbs him, when the stars and planets are favourably aligned, he is able to achieve complexional balance. Of course, it is impossible to infer too much from his short description of his health and mood, yet this passing comment reveals some awareness of astrology and its influence on climate and health.

35 In another letter to Beneš of Hořovice from Paris, Jenštejn briefly reports on his good health. Loserth, ‘Beiträge zur Geschichte der husitischen Bewegung’, epistulum 66, 386–87.

36 For more on miasma and the environment in Bohemia, see David Tomíček, ‘Water, Environment, and Dietetic Rules in Bohemian Sources of the Early Modern Times’, in Albrecht Classen, ed, *Bodily and Spiritual Hygiene in Medieval and Early Modern Literature: Explorations of Textual Presentations of Filth and Water*, Fundamentals of Medieval and Early Modern Culture, vol 19 (Berlin: De Gruyter, 2017), 455. For miasma theory more generally, see Carole Rawcliffe, *Urban Bodies: Communal Health in Late Medieval English Towns and Cities* (Woodbridge: Boydell, 2013), 188–92.

37 Polc, *Česká církev v dějinách*, 302.

38 Claire Weeda, *Ethnicity in Medieval Europe 950–1250: Medicine, Power and Religion*, Health and Healing in the Middle Ages, vol 2 (York: York Medieval Press, 2021), 110; Peter Biller, ‘Proto-Racial Thought in Medieval Science’, in Miriam Eliav-Feldon, Benjamin Isaac and Joseph Ziegler, eds, *The Origins of Racism in the West* (Cambridge: Cambridge University Press, 2009), 163.

39 Joseph Ziegler, ‘Physiognomy, Science, and Proto-racism 1200–1500’, in Miriam Eliav-Feldon, Benjamin Isaac and Joseph Ziegler, eds, *The Origins of Racism in the West* (Cambridge: Cambridge University Press, 2009), 185.

40
*Ibid*., 1200–1500’, 195–96; Biller, ‘Proto-Racial Thought in Medieval Science’, 165–66; Avicenna, *A Treatise on the Canon of Medicine of Avicenna*, Oskar Cameron Grunner (trans) (New York: AMS Press, 1973), 6, 165. See also Gregor Maurach, ed., ‘Johannitius, *Isagoge ad Techne Galieni*’, *Sudhofs Archiv*, 1978, 62, 156.

41 Weeda, *Ethnicity in Medieval Europe*, 114–16. In fact, Jacques Despars (d. 1458) points to the example of, what he terms, Ethiopians in France as proof that one’s complexion could acclimatise to a radically different climate. Ziegler, ‘Physiognomy, Science, and Proto-racism 1200–1500’, 195–96; Jacques Despars, *Commentum*, in *Hic merito inscribe potens vite liber corporalis Abohali Abynsceni canonis libros quinque duflici fere per totum comment munos nuperque translatos … doctors circa textum positi ut locis suis apparebit hi sunt: Gentilis de Fugineo … [et al.], 4 partes* (Venice: Benalius, 1503), f. 22^r^.

42 For instance, Albertus Magnus explains in his *Liber de natura loci* that a traveller must return to their home regularly, otherwise the unfamiliar environment would take its toll on the complexion. Albertus Magnus, *Liber de natura loci* 2.3, in Paul Hossfeld, ed, *Opera omnia*, 40 vols (Aschendorff: Monasterii Westfalorum, 1980), v.ii, 25–27; Weeda, *Ethnicity in Medieval Europe*, 114.

43 Vatican Lat. 1122, ff. 76^r^; PNK, Osek 37, f. 151^r^; Biblioteka uniwersytecka, I F 777, f. 13^r^. All manuscript transcriptions use Vatican Lat. 1122 as the base text. ‘Namque michi contigit, quod enarro gracia studiorum, cum in monte Pessulano degerem ubi esset de mundo nobillimus studium medicorum, et egregij reperiuntur medici. Ibidem infirmari grauiter cepi fere ebdomada integram. Enotabilioribus autem aliis me uisitauit medicus nomine Johannes Jacobi, quem postmodum Parysius Rex Francorum Karolus in eodem ad huc studio me perseuerante propter suam eleganciam, ut sui curam gereret assumpsit.’ Translated in Patrick Outhwaite, *Christ the Physician in Late-Medieval Religious Controversy: England and Central Europe, 1350–1434*, Health and Healing in the Middle Ages 7 (York: York Medieval Press, 2024), 94.

44 Johann Loserth, ‘Beiträge zur Geschichte der Husitischen Bewegung. II. Der Magister Adalbertus Ranconis de Ericinio’, *Archiv für österreichische Geschichte*, 1879, 57, 272.

45 Melissa P. Chase, ‘Fevers, Poisons, and Apostemes: Authority and Experience in Montpellier Plague Treatises’, *Annals of the New York Academy of Sciences*, 1985, 441, 153–170.

46 Vatican Lat. 1122, ff. 76^r–v^; PNK, Osek 37, f. 151^r–v^; Biblioteka uniwersytecka, I F 777, f. 13^v^: ‘Hic cum grauiter decumberem, et ipse de uita mea desperasset, pulmentum michi quodam dari mandauit. Vinum autem omnino prohibuit bibere nisi semel uel bis, idipsum duplo limphatum sed magis me infirmante. Contigit die quadam scolarem quendam michi cibum et potum in absencia aliorum secundum libitum mee uoluntatis dedisse. Cumque uix duo coclearia pulmenti recepissem, octo uero de forti uino ciphos epotaui. Et ex inde sospitatem perfectam recepi. Igitur medicus post paululum ad me uenit uerius manum contactis et succlamare stupefactus cepit: “Sanus est. Sanus est,” nimirumque admirans inquirere cepit, unde michi sanitas euenisset, sibique quod gestum fuit, narrantibus dicere cepit. “Ve nobis! sepe enim falluntur alienigenas domesticis curare medicamentis, qui uita moribus situ longe a nobis distare uidentur.”’ Outhwaite, *Christ the Physician*, 94–95.

47 Lett, ‘*Judicium Medicine* and *Judicium Sanctitatis*’, 166.

48 Vatican Lat. 1122, f. 76^r^; PNK, Osek 37, f. 151^r^. Note that in Biblioteka uniwersytecka, I F 777, a folio is wanting between folios 12 and 13. Hence, this section of the text does not feature. ‘Sane ut frequenter contingit eciam medicos quosdam secundum complexionem propriam omnes alias complexiones indicare. Et idcirco medicinas eis tamquam magis notas et per experimentum comprobatas ceteris conmunicare infirmis.’

49 Vatican Lat. 1122, f. 76^r^; PNK, Osek 37, f. 151^r^; wanting from Biblioteka uniwersytecka, I F 777. ‘Quippe enim similiter est depictore et sculptore ymaginum exemplum, qui proprietas singulares in suis retinent artibus et ymaginibus, quibusdam oblongas quibusdam rotundas alijs crassas et alijs macilentas effigies figurantibus. Ut a discipulis sine arte consimili magistris opus cuiusque artificis discernatur. Unde nimirum infirmitates plurime et mortes propter naturalem medicorum inpressionem et proprietatem ad quam magis inclinantur succedere possunt.’

50 Vatican Lat. 1122, f. 77^r^; PNK, Osek 37, f. 152^r^; Biblioteka uniwersytecka, I F 777, f. 14^r^: ‘In uacuum igitur medicus carnalis laborat quando corpus sanat et uicium anime unde manat infirmitas ignorant, neque ergo dubium medicos id circo falli et fallere posse plurimos. Medicus namque carnalis morbos uel languores, quos uisu et tactu et alijs manifestis experimentis comprehendit; quantum ualet manifestis curare nititur medicamentis. Ea uero que inuisibilia et intangibilia sunt ab exterioribus sensibus non discernuntur. Utpote spiritus et anime internas et inuisibiles passiones, nulla ualent racione conprehendere et similiter curare. Nam in libro sapiente [16: 12] scribitur, hii qui diuersis morbis infecti erant non per hoc quod uidebant saluabantur sed per omnium saluatorem, “neque herba neque malagma sanauit eos, sed sermo domini qui sanat omnia.” Hec ideo subintuli, quia eciam de uisibili morbo sepe oculus et manus fallitur medicorum.’ Outhwaite, *Christ the Physician*, 96–97.

51 Jenštejn may also have had King Asa in mind, who died when he turned to physicians rather than the Lord to cure a violent pain in his feet in II Paralipomenon 16: 12–14.

52 McCleery, ‘Saint, Physician, Diabolical Doctor, Medieval Saint’, 121–22; Jordan, ‘Disappearance of Galen’, 703–13; Jones, *Medicine of the Friars*, 100–2, 136–9; Crisciani, ‘Per una ricerca su “experimentum”-“experimenta”’, 9–49; McVaugh, ‘Nature and Limits of Medical Certitude’, 62-84; Jacquart, ‘Theory, Everyday Practice’, 140-60.

53 Jenštejn misses out ‘occasio praeceps’ in the famous quotation. Quotations of the lines varied in the Middle Ages. For instance, ancient authors tended to write ‘experimentum periculosum’ rather than ‘fallax,’ but other authors such as Arnold de Villanova chose to translate ‘ἡ δὲ πεῖρα σφαλερή’ as ‘experimentum fallax.’ Jenštejn’s word choice and omission may simply be the way it was presented in his exemplar (if he indeed used one rather than relying on his memory) and not necessarily an indication of unfamiliarity with Hippocrates. See Pearl Kibre, ‘Hippocrates Latinus: Repertorium of Hippocratic Writings in the Latin Middle Ages (II)’, *Traditio*, 1976, 32, 257–92; Michael R. McVaugh, ed, *l. Arnaldi de Villanova Expositio super aphorismo Hippocratis «In morbis minus», II. Arnaldi de Villanova Repetitio super aphorismo Hippocratis «Vita brevis»*, Arnaldi de Villanova Opera Medica Omnia, vol 14 (Barcelona: Fundació Noguera/Universitat de Barcelona, 2014); Faith Wallis, ‘Why was the Aphorisms of Hippocrates Re-Translated in the Eleventh Century?’, in Carlos Fraenkel, Jamie Fumo, Faith Wallis and Robert Wisnovsky, eds, *Vehicles of Transmission, Translation and Transformation* (Turnhout: Brepols, 2011), 179–99.

54 Vatican Lat. 1122, f. 76^v^; PNK, Osek 37, ff. 151^v^–152^r^; Biblioteka uniwersytecka, I F 777, f. 13^v^: ‘Ecce quanta dubia et incerta medicamentorum tradicio, et inexperta medicinarum et medicorum sciencia. Prout idipsum experti dicunt medici, et inponitur Ypocrati qui dixit: “Vita breuis, Ars uero longa, Experimentum fallax, Iudicium difficile.” Et quasi concludendo subiungam medicum doctum et expertum experienciam et scienciam habere debere omnium precedencium medicorum; et sic vix adhuc effugiet, quod in multitudine, quam cottidie curat et uisitat, quod in aliquo non offendat et irregularitatem non incurrat. Quia offendens in uno factus est omnium reus, et sic grauiter peccat.’ Outhwaite, *Christ the Physician*, 97–98.

55 This same argument was frequently used against the clergy—namely, that corrupt individuals coloured the way the clergy was received as a whole. For more in a Bohemian context, see Stanisław Bylina, *Wpływy Konrada Waldhausena na Ziemiach Polskich w drugiej Połowie XIV i Pierwszej Połowie XV Wieku* (Wrocław: Zakład Narodowy im Ossolińskich, 1966), especially 24–25; Peter C. A. Morée, *Preaching in Fourteenth-Century Bohemia: The Life and Ideas of Milicius de Chremsir (+1374) and his Significance in the Historiography of Bohemia* (Slakov: EMAN, 1999), 100–26.

56 Vatican Lat. 1122, f. 76^r^; PNK, Osek 37, f. 151^r^; wanting from Biblioteka uniwersytecka, I F 777: ‘Quispiam ne eciam perpendendum, Ypocratem, Galienum, Auicennam et ceteros medicos longinquis a nobis distare regionibus, ubi elementa herbe condiciones hominum et alie circumstancie aliter se habere possunt uel eciam contrarie. Utpote in quibusdam terris plurima abstinencia regi. In quibusdam nimia habundancia homines effluere. Et tamen robustiores et magis longeuos inueniri abstinentibus crapulosos, cum iuxta dictamen rationis recte contrarium debeat pocius esse. Cum ubique ergo non fuerunt propriisque oculis res et alias circumstancias non subiecerint, in suis cur codicibus uasti orbis infirmitas, infirmitates et morbos curare doceant et presumant?’

57 See Pavlína Cermanová, ‘The Apocalyptic background of Hussite radicalism’, in Pavel Soukup and Michael Van Dussen, eds, *A Companion to the Hussites*, ed., Brill’s Companions to the Christian Tradition, vol 90 (Leiden: Brill, 2020), 187–218.

58 Vatican Lat. 1122, f. 76^v^; PNK, Osek 37, f. 151^v^; Biblioteka uniwersytecka, I F 777, f. 13^r^: ‘Iam iamque in finem seculorum deuenimus […], nunc terra nostra uersa est *in salsuginem* succrescente *malicia inhabitancium in ea* [Ps. 106: 34], in qua iam uirtutes herbarum, arborum, fructuum, aromatum, frugum et variorum liquorum inde proueniencium adeo ex inanire cernimus, ut haut nisi parum aut nichil ad sanitatem proficiant. Unde argumentum racioni consonum et conueniens nasci posset veteres tradiciones medicorum que antiquis temporibus profuerunt; utcumque modo pro sanitate nichil uel parum proficere propter antiquitatem temporum, defectum que ampliorem creaturarum et nouissima tempora que omnis finem consumacionis expectant alia medicamenta requirunt.’

59 See, for instance, Jana Nechutová, ‘Reform- und Bußprediger von Waldhauser bis Hus’, in *Kirchliche Reformimpulse des 14./15. Jahrhunderts in Ostmitteleuropa*, ed. Winfried Eberhard and Franz Machilek (Cologne, Weimar, and Vienna: Böhlau, 2006), 239–54.

60 Marie-Christine Pouchelle, *The Body and Surgery in the Middle Ages*, R. Morris (trans) (Oxford: Wiley-Blackwell, 1990), 56, 104; Carole Rawcliffe, *Medicine and Society in Later Medieval England* (Phoenix Mill: Alan Sutton, 1995), 60.

61 Vatican Lat. 1122, f. 76^v^; PNK, Osek 37, f. 151^v^; Biblioteka uniwersytecka, I F 777, f. 13^v^: ‘secundum quosdam varietatem temporum statuta eciam licet immutare et leges nouas condere propter delicta recencia, nouas penas propter diuersas contingencias, diuersa remedia propter egritudinum pluralitatem; nouissime iam etatis medicamenta noua consideratis circumstancijs prolibatis, que in medicine tradicione prudentis per industriam medici legitime ponderantur. Ut salua sic loquar gracia, medicorum expedire antiquos in aliquibus locis codices emendare, corrigere, uel eciam totaliter abolere. Et considerata infirmitate condicionis humane et ceteris, que nouissimo tempore omnem magis possident, hominem nouas pocius fingere medicinas. Sicut scriptum est: *Nouis superuenientibus uetera proicietis* [Lev. 26: 10]. Et que in eorum non reperiuntur canone, herbarum uires et pigmentorum uarias coniuncciones de nouo componere, ut tempori et humane fragilitati se ualeant coaptare. Quod si secus, si aliter faciunt errare; eos quis sapiens haut non nouit. Cum plerique uideant confutatos et a gracia medicorum spretos, infirmos industria laycorum uel muliercularum nouis et inconsuetis herbis et medicamentis aliquando curari.’

62 Macdougall, ‘Surgeon and the Saints’, 260.

63 Irma Taavitsainen, *Middle English Lunaries: A Study of the Genre,* Mémoires de la Société Néophilologique de Helsinki, vol 47 (Helsinki: Société Néophilologique, 1988), 116.

64 Rawcliffe, *Urban Bodies*, 300; Carole Rawcliffe, *Leprosy in Medieval England* (Woodbridge: Boydell Press, 2006), 249.

65 Irma Taavitsainen, ‘Changing Conventions of Writing: The Dynamics of Genres, Text Types, and Text Traditions’, *European Journal of English Studies*, 2001, 5, 139–150. A prime example of the type of text that was widely used across Europe is the *Circa instans*, a pragmatic pharmacological book that provides entries on different plants, herbs and minerals used in medicines. In fact, this work itself was often altered and adapted to suit the needs of the practitioner. See Iolanda Ventura, ‘Il *Circa instans* attribuito a Platearius: trasmissione manoscritta, redazioni, criteri di costruzione di un’edizione critica’, *Revue d’Histoire des Textes*, 2015, 10, 249–362.

66 Vatican Lat. 1122, f. 78^r^; PNK, Osek 37, f. 153^r^; Biblioteka uniwersytecka, I F 777, f. 15^r^: ‘Ipse denique salutaris sanctus est nobis medicus et salutare medicamentum, quia ipse cognouit figmentum nostrum pro eterna namque sanitate nostra non requirebamus ydoneum alium, sicut [Isaiah 53: 3] “uirum dolorum et scientem infirmitatem.”’ Outhwaite, *Christ the Physician*, 98–99.

67 Michael R. McVaugh, ‘Bedside Manners in the Middle Ages’, *Bulletin of the History of Medicine*, 1997, 71, 201–33; Mary Catherine Welborn, ‘The Long Tradition: A Study in Fourteenth-Century Medical Deontology’, in James L. Cate and Eugene N. Anderson, eds, *Medieval and Historiographical Essays in Honour of James Westfall Thompson* (Chicago: University of Chicago Press, 1938), 344–57.

68 Włodzimierz Zega, *Filozofia Boga w* Quaestiones sententiarum *Mikołaja Bicepsa: krytyka prądów nominalistycznych na Uniwersytecie Praskim w latach osiemdziesiątych XIV wieku* (Warsaw and Bydgoszcz: IFiS PAN, 2002), 42–47; Vielhaber, ‘Der *Libellus de bono mortis* des Erzbischofs Johann von Jenstein’, 163.

69 Vatican Lat. 1122, f. 75^r^; PNK, Osek 37, f. 150^r^: ‘Namque cena nobiscum peracta unusquisque ad propria remeauit, et illa nocte omnes eramus infirmi per plures dies. Ipse tamen magister epydemie morbo grauiter sancius uix duobus diebus et noctibus superuixit.’

70 Martin Dekarli makes a similar point about Jenštejn’s manipulation of anecdotes for political ends. *Wyclif, Páleč a nominalisté na pražské univerzitě v pozdním středověku*, Středověk, vol 7 (Prague: NLN—Nakladatelství Lidové noviny, 2023), 66–67.

71 For a political history of this journey, see Rudolf Holinka, *Církevní politika arcibiskupa Jana z Jenštejna za pontifikátu Urbana VI* (Bratislava: Filosofická fakulta University Komenského, 1933), 39–49.

72 This change of route seems to contradict his advice given to Beneš of Hořovice in the same year that moving to avoid the plague was ill advised. The only qualifier we might add is that Jenštejn was travelling for diplomatic reasons and not relocating as Beneš was suggesting. Loserth, ‘Beiträge zur Geschichte der husitischen Bewegung’, epistulum 70, 389: ‘Qui prius sauciato corpore menbrisque adeo debilibus ad desperacionem usque medicorum perveneram, divina gracia disponente, quod phisicorum negabat collirium, hoc grate omnipotentis sanavit ungentum, ita quod in sanitate bona corporis et robore a Colonia gressus meos direxi usque in Nuremberg civitatem […]’.

73 Loserth, ‘Beiträge zur Geschichte der husitischen Bewegung’, epistulum 5, 309. In a letter to the Superior General of the Dominican Order, Raymond of Capua (d. 1399), he explains: ‘Namque febre ingenti adeo seviente infelix vita nutabat, salutis spes aberat, nos phisici morituros acclamabant. Sic longis attriti febribus macieque consumpti diutina gravissimi paracismi dominarunt omnia ossa nostra’.

74 Polc, *Česká církev v dějinách*, 299–300 n. 35, which corrects the transcription from Loserth, ‘Beiträge zur Geschichte der husitischen Bewegung’, epistulum 71, 390: ‘octo enim integris saeptimanis febris nos agitabat pre nimia et duplici contricione conterebat, videlicet duplici terciana, quam singulis dies habuimus, que iam algore nimio corpus nostrum impie perturbabat ita ut nos valido tremore frigus quateret, iam fervidissimo torrebat incendio, ut crederemur ignibus consumi rapacibus, ita quippe, quod vigore exhausti, consueto robore privati atque macie consumpti nervorum nostrorum numerum suum aspicientibus ostentabant.’

75
*Vita domini Iohannis*, ed. Krmíčková, 444.

76 Polc, *Česká církev v dějinách*, 299–300 n. 35; from Loserth, ‘Beiträge zur Geschichte der husitischen Bewegung’, epistulum 71, 390: ‘Sed tamen Matris gloriose presidio, cuius tunc dies erat, Omnipotentis misericordia adiuti, qui post virgam coreccionis baculum porrigit sue clementis pietatis, dimissa febre corpus debile paulisper iterum ignem naturalis caloris resumpsit artusque nostri forcius de die in diem solidando, cutis vacua molem videtur carnem assumere, nervi quoque reassumpto; vitalium spirituum robore sub carnis crassitudine delitescunt, ita quod ex Dei munere aliud non restat quam sanitas.’

77 Jaroslav Kadlec, ‘Začátky kláštera augustiniánských kanovníků v Roudnici’, *Studie o rukopisech,* 1981, 20: 65–86.

78 Holinka, *Církevní politika arcibiskupa Jana z Jenštejna*, 61–62.

79 Polc, *Česká církev v dějinách*, 300.

80 Kadlec, ‘Petr Klarifikátor, duchovní vůdce a životopisec arcibiskupa Jana z Jenštejna’, 101–50.

81 For more on the feast, see Weltsch, *Archbishop John of Jenstein*, 88–91; Michael Van Dussen, *From England to Bohemia: Heresy and Communication in the Later Middle Ages* (Cambridge: Cambridge University Press, 2012), 46–48; Jaroslav V. Polc, *De origine festi visitationis B.M.V.* (Rome: Pontificia Università Lateranense, 1967); idem, ‘La Festa della Visitazione e il Giubileo del 1390’, *Rivista di Storia della Chiesa in Italia*, 1975, 29: 149–72.

82 Sedlák, *M. Jan Hus*, 47*–48*: ‘nam post salutarem medicum et salubre medicamentum, quod in Christi corpore sumpseram, carnales medici inportunis admonicionibus instabant, ut medicinam corporalem sumerem. Quibus aiebam alia me non agere nisi ea que sanant. Cogitabam enim in animo meo alias michi non expedire sumere ulterius medicinas, si perfecte sanari volo; quodsi secus facerem, pro certo hoc scire me in priorem relabi egritudinem. Sed perseverante medico et instante cum aliis, ut aliquid pro sustentacione sumerem, ego miser et fragilis proclivis ad malum parui, ut obediens fierem et ne medicos spernerem, qui de sanitate mea errant solliciti; confortacionem corporalem recepi et confortacionem spiritualem quantum ad corporis sospitatem amisi.’

83 Vatican Lat. 1122, f. 77^v^; PNK, Osek 37, f. 152^v^; Biblioteka uniwersytecka, I F 777, f. 14^v^: ‘Lucas uidelicet medicus cuius laus in euangelio, gloriosi martires Cosmas et Damianus beatus eciam Panthaleon cum ceteris sanctis qui utroque modo diuina suffulti gracia, et corporis et anime medici exstiterunt quamquam magis anime quam corporis, animam oratione deuota.’ Outhwaite, *Christ the Physician*, 99.

84 Richard Rutt, ‘Saints Cosmas and Damian: Patron Saints of Medicine. A Story from Prayers and Pictures?’, *Journal of Medical Biography*, 1994, 2, 48–52.

85 Rawcliffe, *Urban Bodies*, 299.

## Author Biography

Patrick Outhwaite is an Assistant Professor in the Department of English Language and Culture at the University of Groningen. His research addresses health, religion and literary culture in late-medieval society, with a particular focus on England and Central Europe. In 2024 he published his first monograph, *Christ the Physician in Late Medieval Religious Controversy: England and Central Europe 1350-1434* (York Medieval Press). He is currently undertaking a VENI (Dutch Research Council) project, ‘Licensing “Lechecraft”: Medical Regulation and Patient Scepticism’ (VI.Veni.221C.045).

